# 4D CT to assess spinal instability in developmental anomaly of posterior arch of atlas

**DOI:** 10.1259/bjrcr.20210038

**Published:** 2022-01-20

**Authors:** Stefanie WY Yip, James F Griffith, Ryan KL Lee, King Lok Liu

**Affiliations:** 1 Department of Imaging and Interventional Radiology, Prince of Wales Hospital, The Chinese University of Hong Kong, Shatin, Hong Kong

## Abstract

Four-dimensional (4D) CT uniquely allows cinematic visualization of the entirety of joint motion throughout dynamic movement, which can reveal subtle or transient internal joint derangements not evident on static images. As developmental anomalies of the posterior arch can predispose to cervical spinal instability and neurological morbidity, precise assessment of spinal movement during motion is of clinical relevance. We describe the use of 4D-CT in a subject with partial absence of posterior C1 arch. This, to our knowledge, is the first such report. In at-risk individuals, 4D-CT has the potential to enable an assessment of spinal instability with a higher level of clarity and, in this sense, its more routine implementation may be a future direction.

## Case presentation

An 11-year-old girl with hypoplasia of the posterior arch of C1 presented with neck pain for four years. She did not have any neurological symptoms and enjoyed normal development in all other aspects. Physical examination was unremarkable.

## Investigations

Dynamic 4D non-contrast CT (GE Revolution 256, United States, 100kV, 100mA) of the upper cervical spine was performed with multislice axial images acquired during a single gantry run without table motion. The scan technique involved placing the patient in a lateral-decubitus position while she was instructed to slowly and continuously move her neck between full flexion and extension twice during each gantry rotation. This neck movement was first practiced by the patient in a lateral-decubitus position under supervision on the CT gantry table before scanning commenced. The volume dataset acquired enabled multiplanar bone and soft tissue reconstruction, generating a cine movie clip (Supplementary Video 1) showing 360° view of cervical spinal motion throughout the two cycles of flexion and extension. The upper cervical spinal cord could be readily identified on soft tissue windows.

Supplementary Video 1.

**Figure 1.**
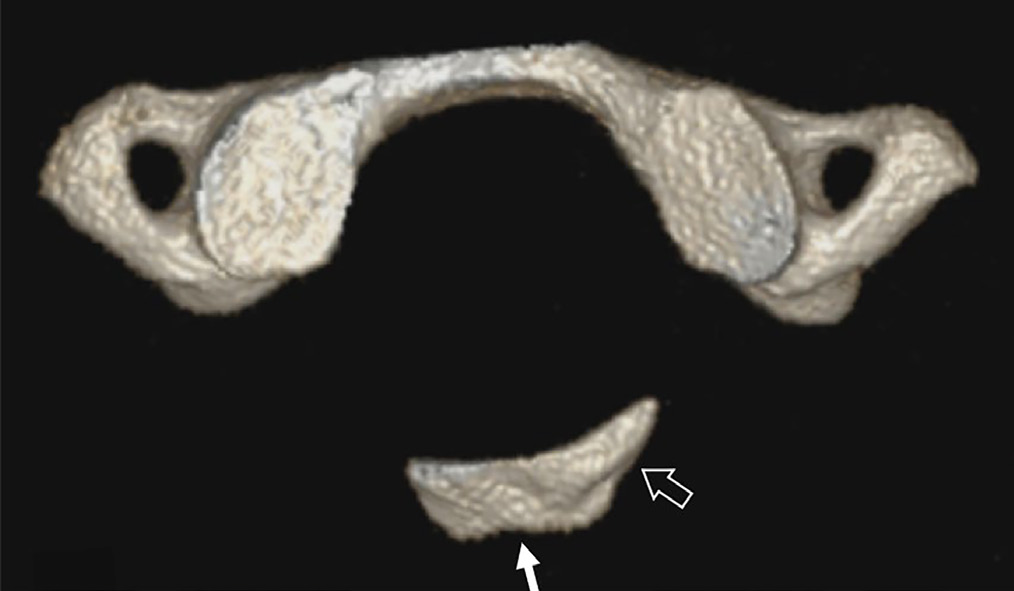
3D reformatted CT static image showing hypoplasia of the posterior arch of atlas with a persistent posterior tubercle (arrow) contiguous with a small left paramedian posterior arch remnant (open arrow), compatible with a Currarino type D posterior arch anomaly.

**Figure 2.**
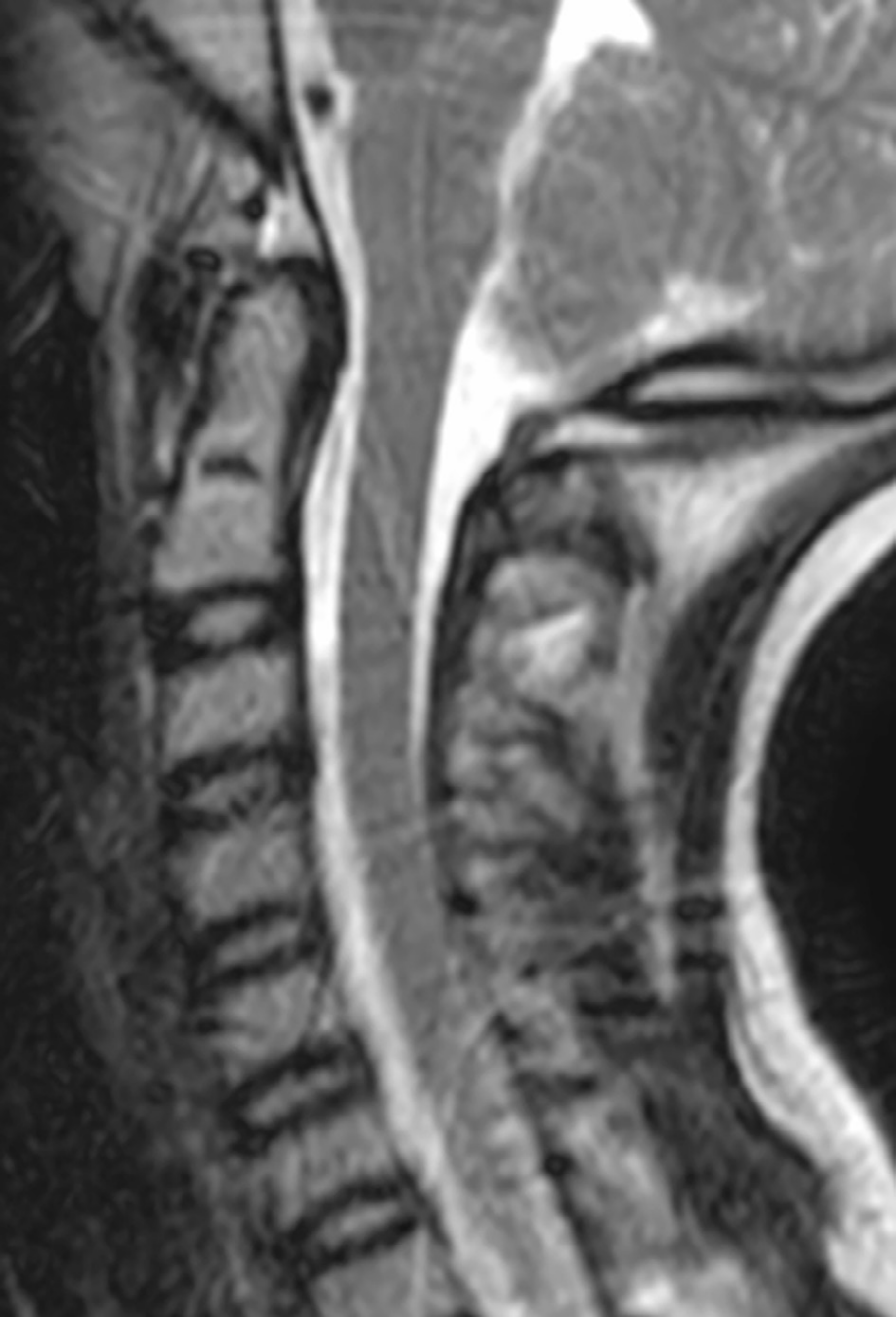
Sagittal *T*_2_-weighted static MR image of the cervical spine at neutral position showing normal alignment with no cord impingement or compression.

Axial reformatted CT in the neutral position showed partial absence of the posterior arch of the atlas with a posterior tubercle contiguous with a small left paramedian posterior arch remnant (Figure 1). The anterior arch and transverse foramina of C1 were normal, that is Currarino type D posterior arch anomaly.^1^ In the neutral position, the persistent posterior tubercle of C1 was normally aligned to the skull base and axis (C2) (Figure 2a).

**Figure 3.**
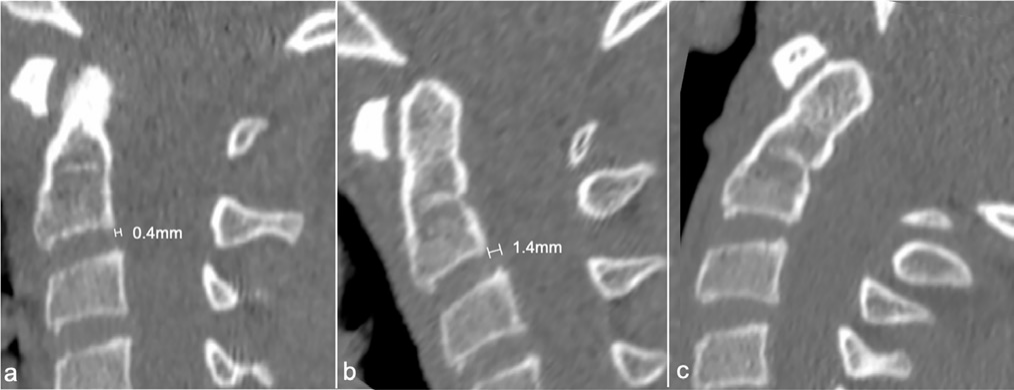
Sagittal CT reconstructed images of the upper cervical spine obtained from the 4D cine. (**a**)Neutral position, showing normal alignment of the craniocervical junction and minimal (0.4 mm, 2%) anterolisthesis of C2 on C3. (**b**)Flexion position showing normal alignment of the C1 posterior tubercle and slight increase in the C2/C3 anterolisthesis to 1.4 mm (7%). (**c**)Extended position showing no change in the alignment of the C1 posterior tubercle and resolution of the C2/3 anterolisthesis.

Quantitative analysis of the captured cine files in sagittal, coronal, and axial planes during flexion and extension showed normal atlantoaxial alignment with no significant displacement of the non-fused posterior elements and no change in spinal canal dimension throughout the range of neck movements. The spinal cord was not compressed throughout the range of neck movement. Minimal (0.4 mm, 2%) anterolisthesis of C2 on C3 was present in the neutral position (Figure 3a), which increased slightly to 1.4 mm (7%) during flexion (Figure 3b), and resolved with no measurable spondylolisthesis during extension (Figure 3c). This mild anterolisthesis was most likely physiological. There was no neural foraminal nor spinal canal narrowing evident throughout the range of movement. The 4D-CT examination enabled a clear assessment of cervical spine movement during the entirety of flexion/extension motion, revealing a subtle increase in malalignment that was not readily evident on static images alone (video).

Static magnetic resonance imaging (MRI) in the neutral, flexed, and extended positions performed on the same day showed no change in the spinal canal dimension at C1 and no cord encroachment or compression at fixed endpoints of maximum neck flexion and extension (Figure 2).

## Outcome

The patient was managed conservatively with no specific treatment and continued to enjoy normal activity with no subsequent complications encountered.

## Discussion

4D-CT is an emerging technological advance that involves acquiring 3D-CT volume data over a period during which the subject performs controlled motion within the scanner, enabling multiplanar reconstruction into a high-resolution cine CT that can display continuous joint motion throughout a range of dynamic movement.^1^ This functional element provides additional information compared to radiographs and MRI obtained in fixed flexion and extension positions, as these non-functional investigations, while providing static images allowing valuable assessment at fixed endpoints, may potentially overlook instability during the dynamic range of motion. There are emerging reports advocating 4D-CT for functional assessment in patients with acquired cervical spinal pathology.^2^

Radiography is a two-dimensional protectional technique in which visualization of the cranio-cervical junction is relatively hindered by overlapping bone and soft tissue structures. Dynamic fluoroscopy is feasible, but similar to radiography, is somewhat limited in its assessment by overlapping bone. Hybrid imaging, such as superimposing 3D-CT images onto real-time fluoroscopic imaging, can create a 4D-element and is currently used to improve localization in interventional procedures,^3^ but the low resolution of fluoroscopic imaging would limit its value in precisely assessing joint movement. Kinematic MRI of the cervical spine is also feasible^4,5^ with good resolution of the spinal cord, but MRI has poorer bone resolution than CT and does not have a 3D component. In addition, the MR neck coil limits the range of neck movement, potentially precluding assessment of the end range of movement that may be most prone to instability.

Developmental anomalies of the posterior arch of the atlas are relatively common^6^ and may predispose to increased risk of neurological morbidity.^7^ In congenital partial absence of C1 posterior arch, the presence of a persistent posterior tubercle, indicating Type C or D Currarino anomaly, places one at higher risk of C1 arch stenosis than other more benign subtypes of posterior arch defect.^8^ This is of particular relevance during dynamic movement, as cord impingement by the non-fused posterior tubercle, only occurring during neck extension is documented.^9^

In our patient who has a Type D Currarino posterior arch anomaly, analysis of the reconstructed cine movie from 4D-CT enabled precise assessment of the relationship between the non-fused posterior tubercle and anterior arch throughout flexion and extension, thus enabling a more confident diagnosis of posterior tubercle stability throughout dynamic motion to be made (video). Should instability have existed, 4D-CT would most likely have enabled precise quantification of the degree of instability, as shown in our illustrative case where a 0.4mm C2/3 anterolisthesis in the neutral position was seen to increase to 1.4 mm on flexion and subsequently resolved on extension. Soft tissue CT window settings were also able to clearly show the outline of the cervical cord, helping to exclude any cord impingement. The principal disadvantage of 4D-CT is the higher radiation dose, which can be 2–4 times higher than that of conventional CT.^10^ However, lower dose volumetric acquisition is possible, and this has been implemented in 4D-CT imaging of pediatric airway disorders.^1^

The dynamic visualization of vertebral motion provided by 4D-CT is potentially of more widespread clinical benefit than represented by this single case. 4D-CT could potentially be used to assess patients with suspected cervical spinal instability. Small intervertebral movements of the cervical spine not evident on static positioning may have significant clinical implications, particularly in the developmentally narrow spinal canal.

## Learning points

- Developmental anomalies of posterior arch predispose to cervical spinal instability.
- A patient with congenital hypoplasia of the posterior arch of C1 is presented for whom 4D-CT enabled a diagnosis or exclusion of spinal instability to be made with high clarity by revealing intervertebral motion throughout the entire range of flexion / extension movement.
- 4D-CT may have a wider role in assessing cervical spinal instability in at-risk individuals, such as those with congenital spinal anomaly.

## References

[1] Kwong Y, Mel AO, Wheeler G, Troupis JM. Four-dimensional computed tomography (4dct): a review of the current status and applications. J Med Imaging Radiat Oncol 2015; 59: 545–54. doi: 10.1111/1754-9485.12326 26041442

[2] Lim KZ, Troupis JM, Daly C, Oehme D, Goldschlager T. Indications for use of dynamic four-dimensional computed tomography in diagnosing instability in spinal cervical conditions. Global Spine Journal March 24, 2017; 6: s-0036. RESOURCE> .. doi: 10.1055/s-0036-1582751

[3] Nobre C, Oliveira-Santos M, Paiva L, Costa M, Gonçalves L. Fusion imaging in interventional cardiology. Rev Port Cardiol (Engl Ed) 2020; 39: 463–73: S0870-2551(20)30285-7. doi: 10.1016/j.repc.2020.03.014 32736908

[4] Tamai K, Buser Z, Paholpak P, Sessumpun K, Hsieh PC, Nakamura H, et al. MRI kinematic analysis of t1 sagittal motion between cervical flexion and extension positions in 145 patients. Eur Spine J May 2018; 27: 1034–41. doi: 10.1007/s00586-017-5385-z 29128915

[5] Jiang X, Chen D, Lou Y, Li Z. Kinematic analysis of cervical spine canal diameter and its association with grade of degeneration. Eur Spine J July 2016; 25: 2166–72. doi: 10.1007/s00586-016-4624-z 27236657

[6] Khanna R, Smith ZA, Dlouhy BJ, Dahdaleh NS. Complete absence of the posterior arch of c1: case report. J Craniovertebr Junction Spine 2014; 5: 176–78. doi: 10.4103/0974-8237.147090 25558151 PMC4279283

[7] Guenkel S, Schlaepfer S, Gordic S, Wanner GA, Simmen H-P, Werner CML. Incidence and variants of posterior arch defects of the atlas vertebra. Radiol Res Pract 2013; 2013: 957280. 10.1155/2013/957280 24109510 PMC3784273

[8] Gangopadhyay S, Aslam M. Posterior arch defects of the atlas: significance in trauma and literature review. Eur J Emerg Med 2003; 10: 238–40. doi: 10.1097/00063110-200309000-00017 12972904

[9] Klimo P, Blumenthal DT, Couldwell WT. Congenital partial aplasia of the posterior arch of the atlas causing myelopathy: case report and review of the literature. Spine 15, 2003; 28: E224-8. doi: 10.1097/01.BRS.0000065492.85852.A9 12811285

[10] Noid G, Tai A, Chen G-P, Robbins J, Li XA. Reducing radiation dose and enhancing imaging quality of 4dct for radiation therapy using iterative reconstruction algorithms. Adv Radiat Oncol 2017; 2: 515–21. doi: 10.1016/j.adro.2017.04.003 29114620 PMC5605285

